# Supplementary figures and images for: PD-1 is imprinted on cytomegalovirus-specific CD4+ T cells and attenuates Th1 cytokine production whilst maintaining cytotoxicity
Source: PLoS Pathog. 2021 Mar 4;17(3):e1009349. doi: 10.1371/journal.ppat.1009349 (PMC7963093; doi:10.1371/journal.ppat.1009349)

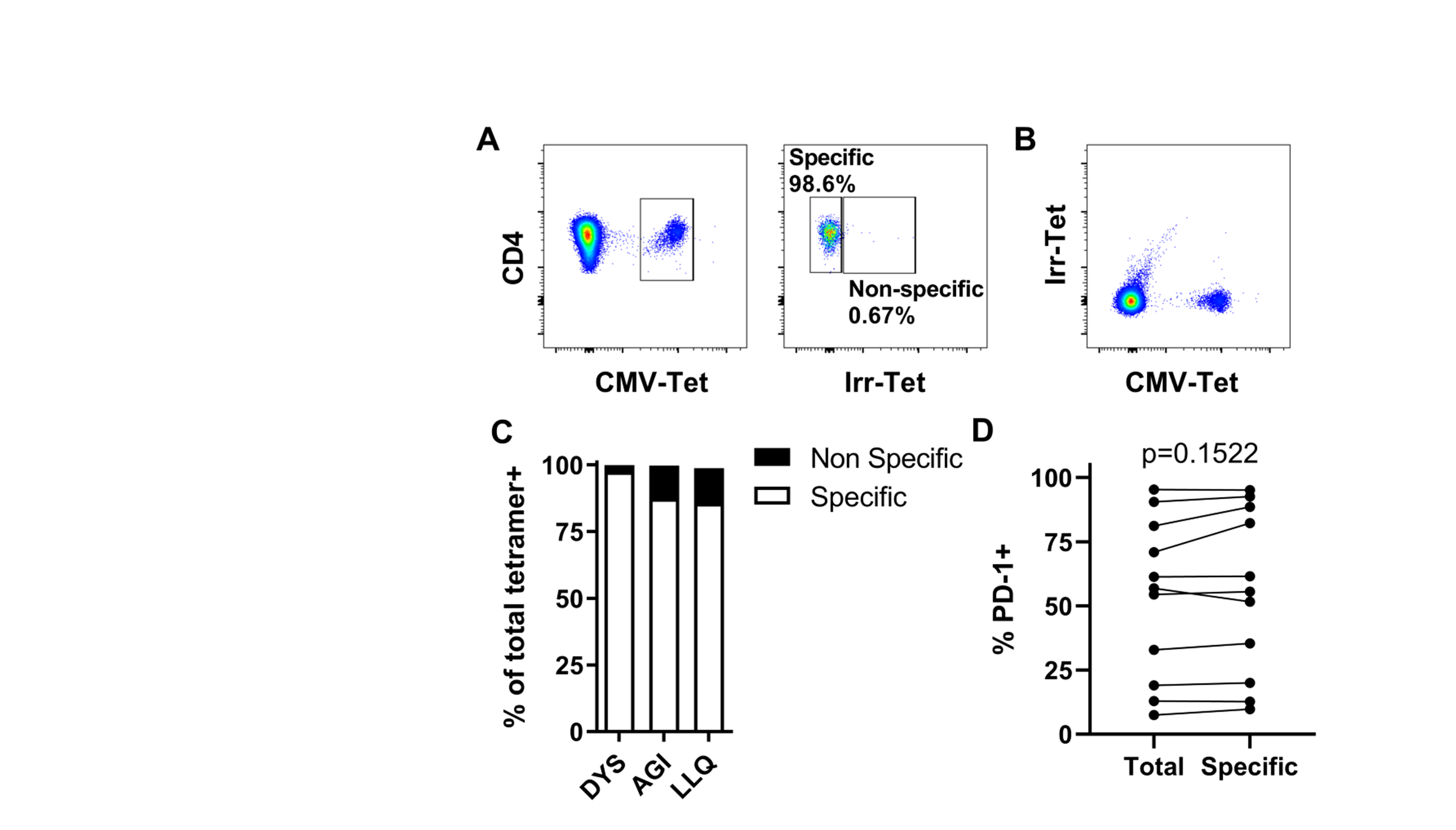

Supplement: S1 Fig — Non-specific tetramer binding was assessed by co-staining with an irrelevant tetramer. (A) Gating strategy used to determine non-specific binding. Tetramer-positive cells were gated (left) and the binding of irrelevant tetramer (Irr Tet) assessed in this population (right). (B) An example of staining of tetramer and irrelevant tetramer in bulk CD4 T cells. (C) Shows summary data for individual tetramers (DYS n = 4, AGI n = 3, LLQ n = 4). (D) The percentage of PD-1+ cells was assessed in the total population of tetramer-binding cells and also in the ‘specific’ population from which the population of irrelevant-tetramer binding cells had been removed. No significant difference was observed in relation to the percentage of PD-1 expression on these two populations (paired t-test). (TIF) [file ppat.1009349.s001.tif]

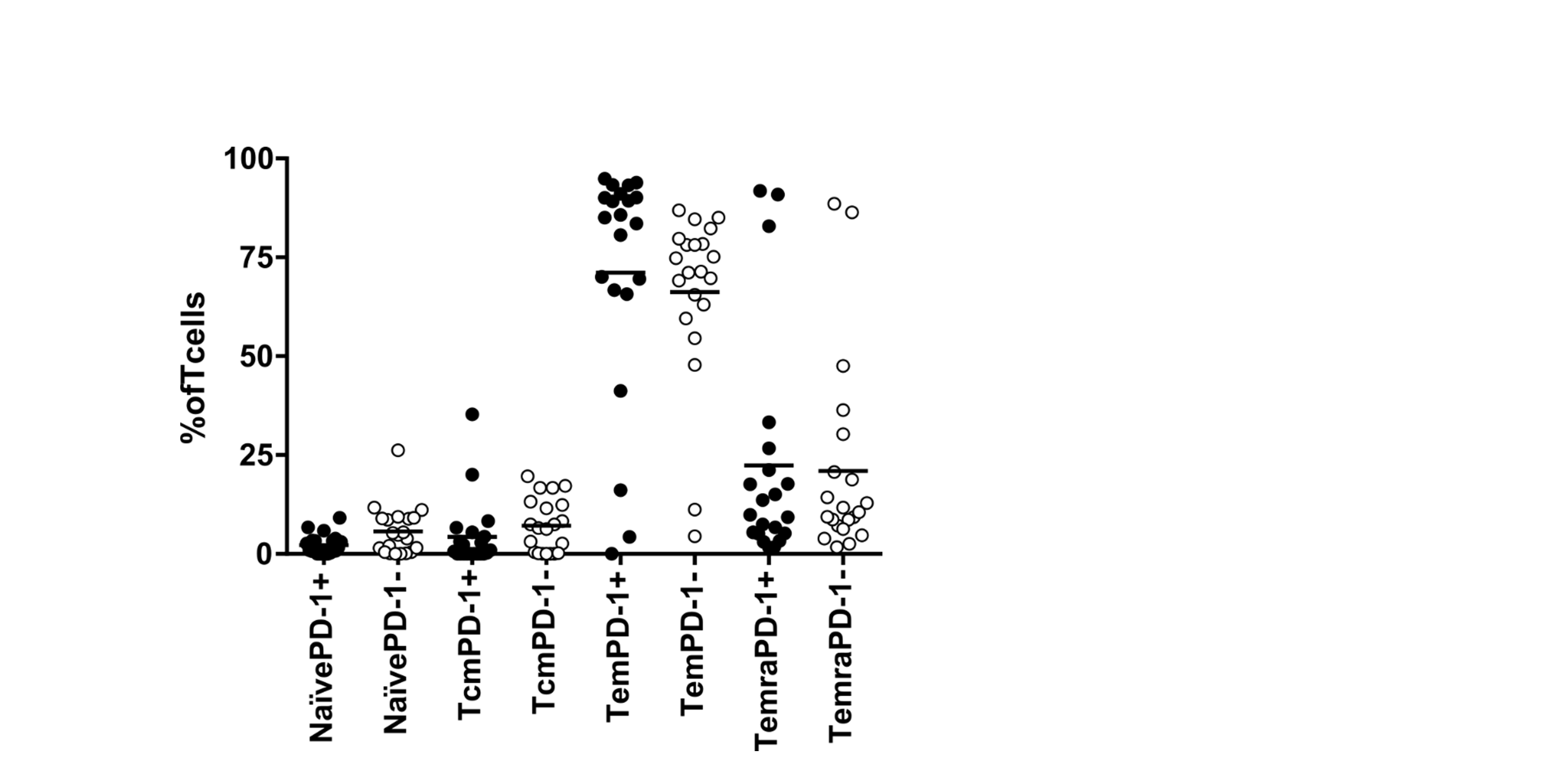

Supplement: S2 Fig — The memory status of PD-1+ and PD-1- CMV Tetramer+ CD4+ T cell subsets was determined on healthy donors (n = 21) by expression of CD45ra and CCR7; Naïve—CD45ra+CCR7+, Tcm -CD45ra-CCR7+, Tem—-CD45ra-CCR7-, Temra—CD45ra+CCR7-. (TIF) [file ppat.1009349.s002.tif]

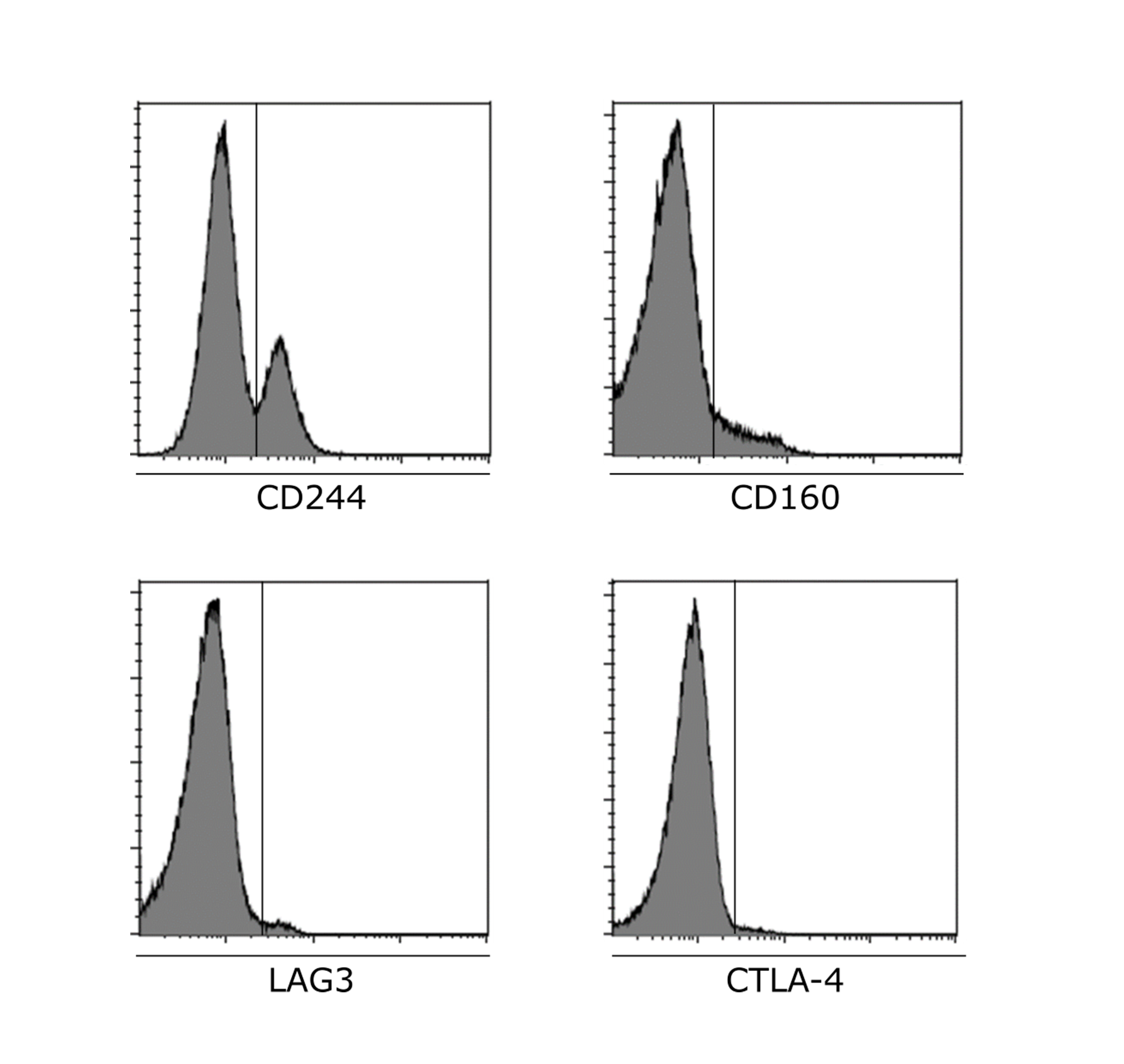

Supplement: S3 Fig — Example plots of the expression of inhibitory receptors on CMV tetramer-specific CD4+ T cells. (TIF) [file ppat.1009349.s003.tif]

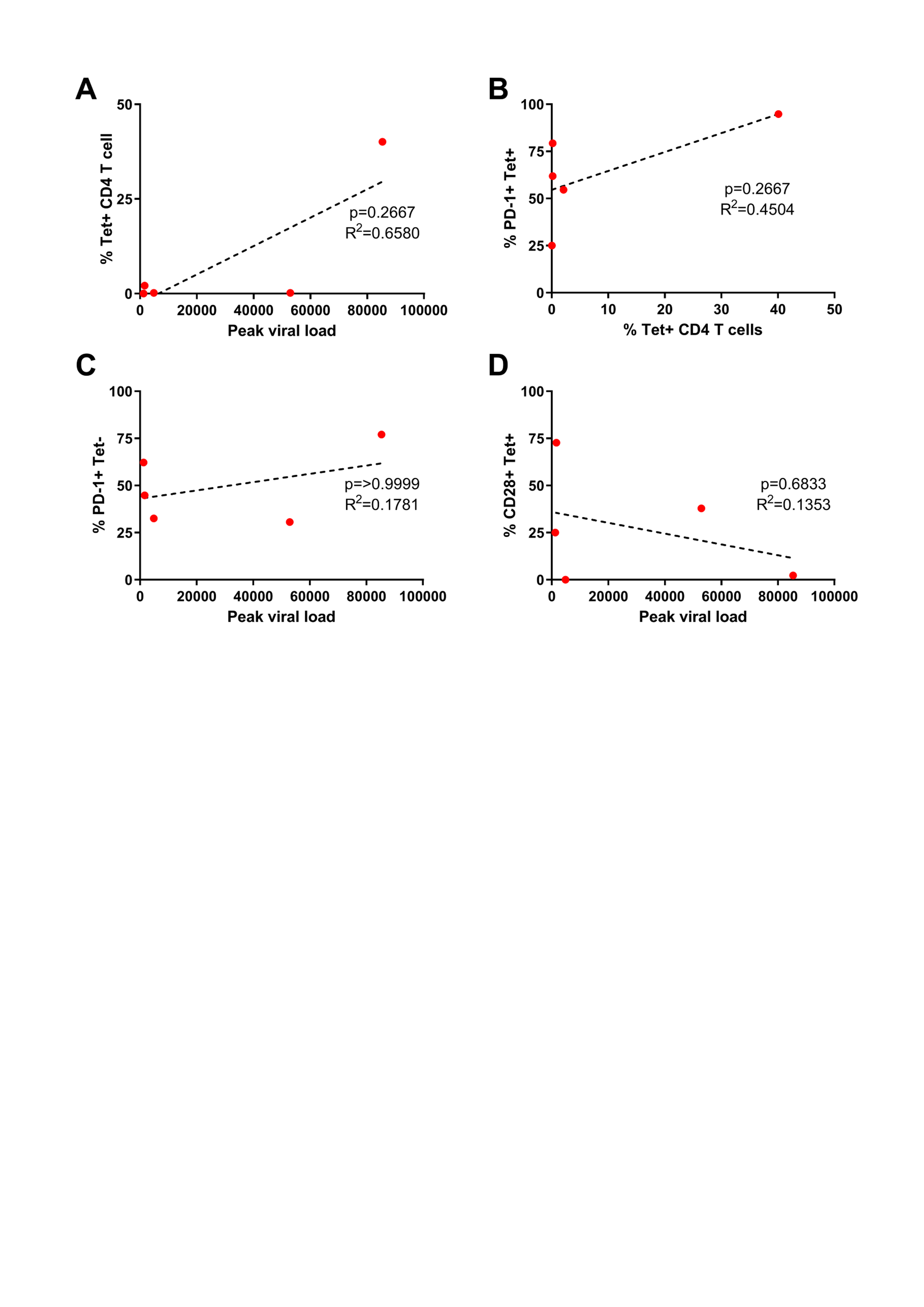

Supplement: S4 Fig — Correlation of parameters from CMV acute infection in bone marrow transplant patients (n = 5) at 25 weeks post resolution of viremia. (A) Peak CMV viral load and frequency of Tetramer+ CD4 T cells. (B) Frequency of PD-1+ Tetramer+ CD4 T cells and Tetramer+ CD4 T cell frequency. (C) Frequency of PD-1+ Tetramer- CD4 T cells and Peak CMV viral load. (D) CD28+ Tetramer+ cells and Tetramer+ CD4 T cell frequency. No correlation was observed. (TIF) [file ppat.1009349.s004.tif]

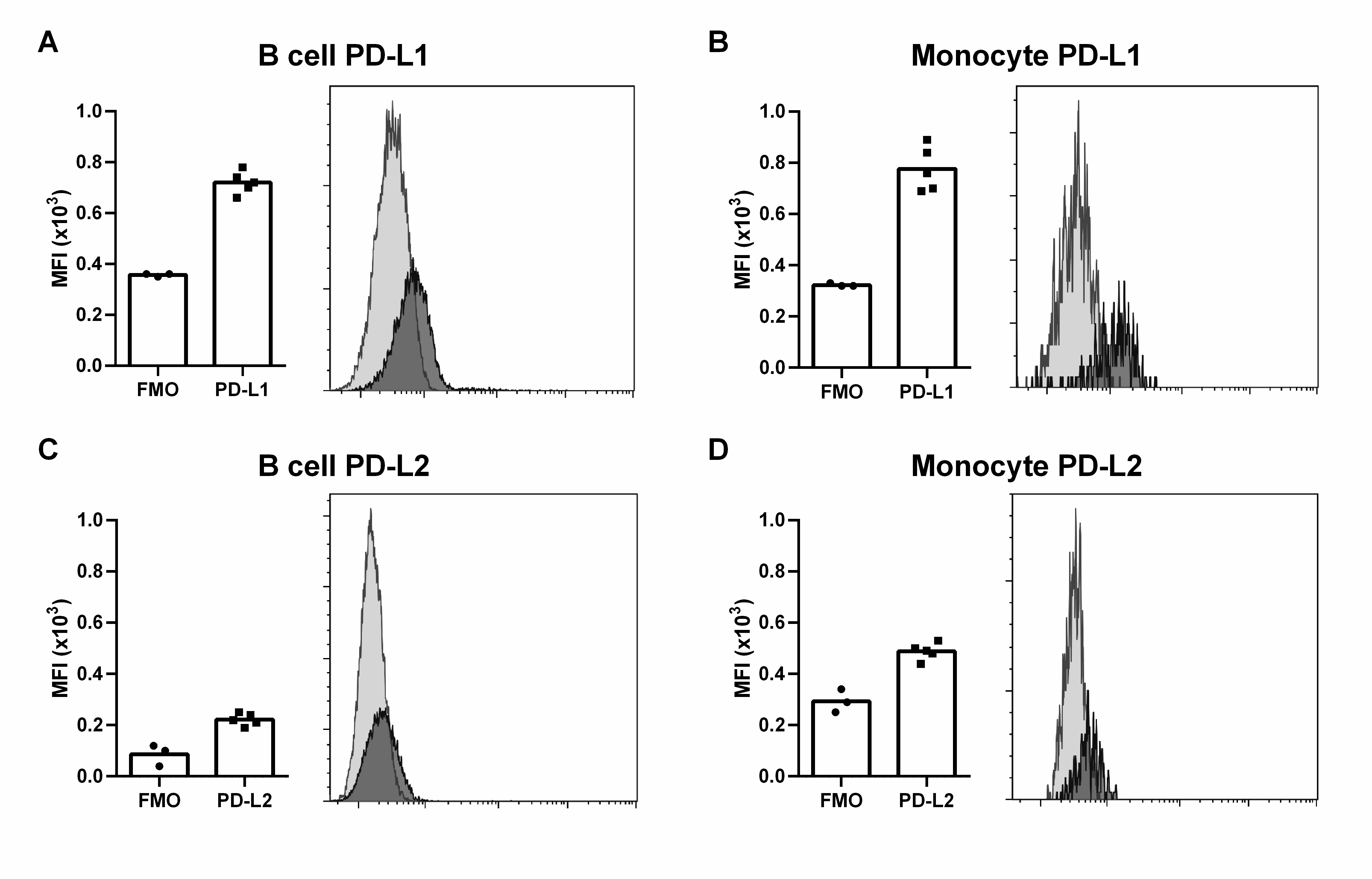

Supplement: S5 Fig — Expression of the PD-1 ligands PD-L1 and PD-L2 was assessed on monocyte (CD14+CD19-CD3-), B cell (CD19+CD14-CD3-) and T cell (CD3+ CD14-CD19-) populations. Expression was found consistently on monocyte and B cell populations. Shown are example plots and combined data from five healthy donors. (TIF) [file ppat.1009349.s005.tif]

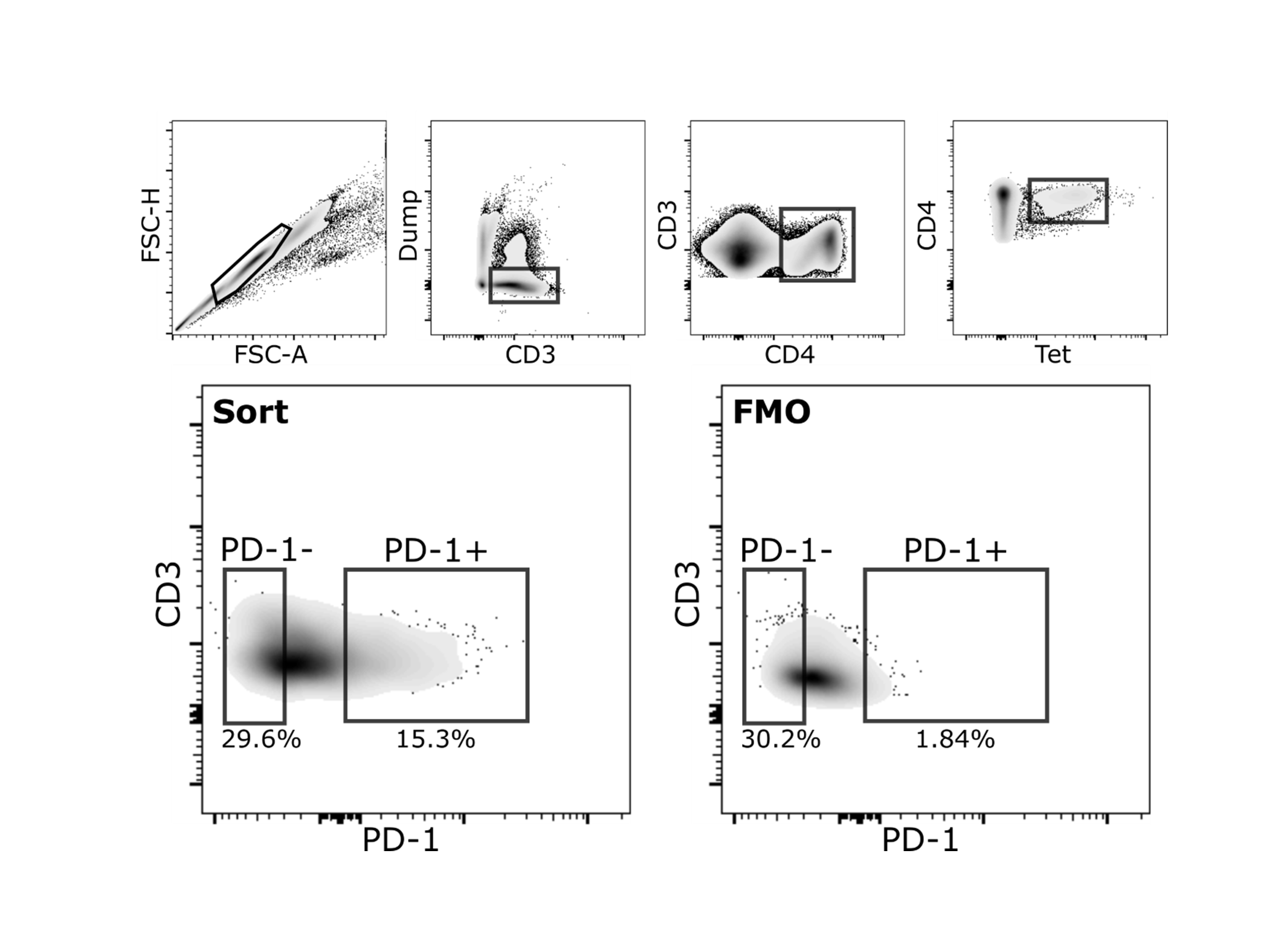

Supplement: S6 Fig — This was used for cell sorting for T cell cloning, single cell TCR sequencing and RNA-seq experiments. (TIF) [file ppat.1009349.s006.tif]

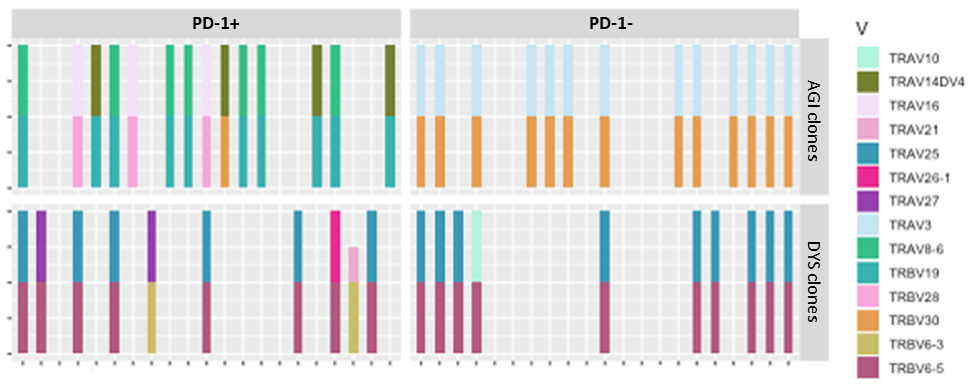

Supplement: S7 Fig — PD-1+ and PD-1- DYS and AGI specific CD4+ T cells cloned by limited dilution ex vivo were single cell sorted. Shown are TCRA and TCRB sequencing of individual T cell clones. No clonotypes are shared between AGI PD-1+ and PD-1- T cell clones, whereas DYS clones show shared TCR usage between PD-1+ and PD-1- T cell clones. (TIF) [file ppat.1009349.s007.tif]

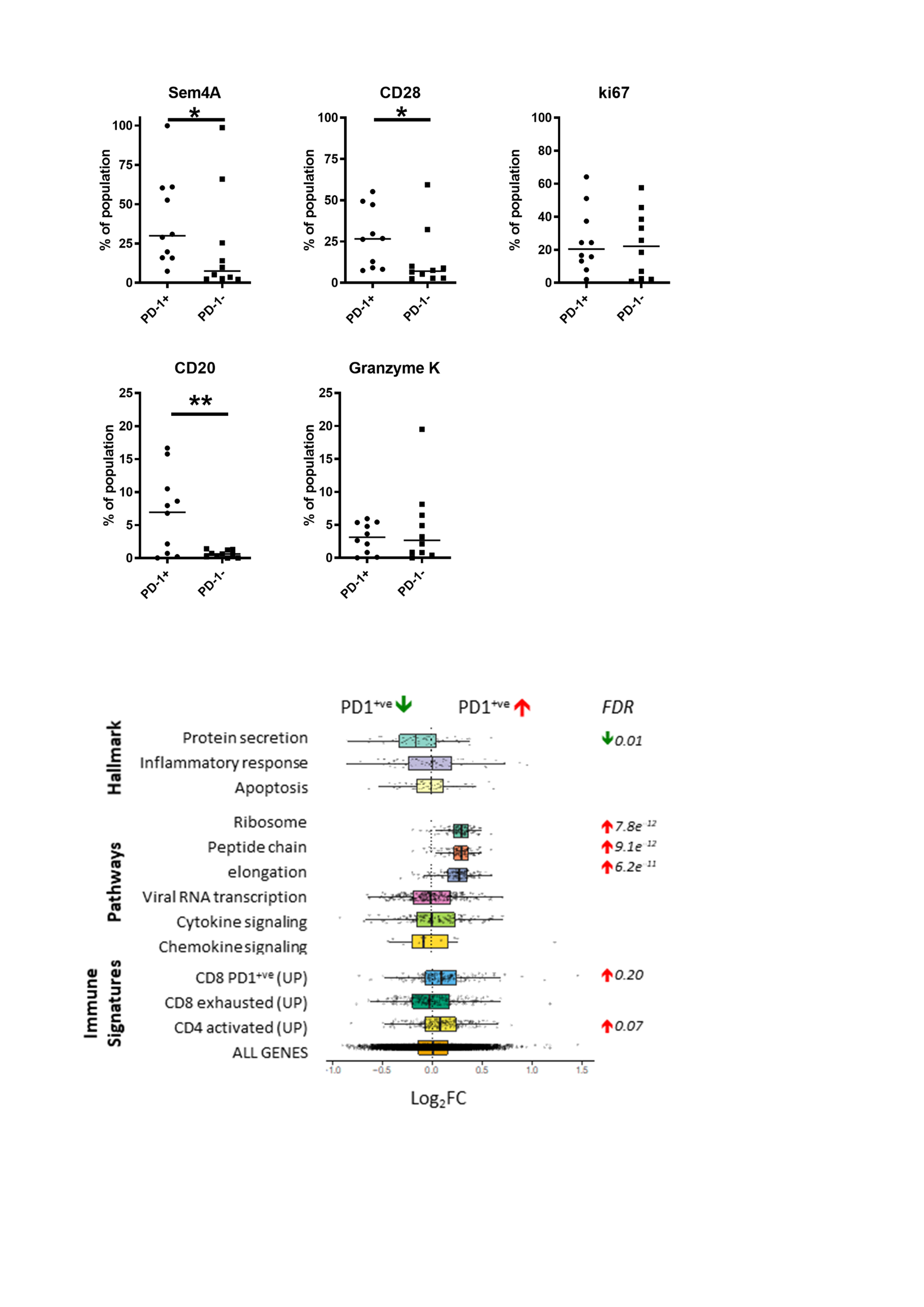

Supplement: S8 Fig — (A) Flow cytometry was used to assess protein expression of a selection of differentially regulated genes on PD-1+ and PD-1- CMV tetramer-positive CD4+ T cells. P values were calculated by two tailed paired t test, * p = <0.05 ** p = <0.005. (B) Selected gene sets were analysed for their enrichments within ranked genes from differential expression analysis between PD1+ vs PD1- CMV tetramer+ CD4+ T cells. Points indicate the log2 fold change (PD1+/PD1-) in expression of genes within the gene set. GSEA false discovery rate (FDR) is reported for gene sets with FDR < 0.3 indicating their coordinated upregulation (↑) or downregulation (↓) in PD1+ cells. (TIF) [file ppat.1009349.s008.tif]
