# Supplementary material for: PD-1 is imprinted on cytomegalovirus-specific CD4+ T cells and attenuates Th1 cytokine production whilst maintaining cytotoxicity
Source: PLoS Pathog. 2021 Mar 4;17(3):e1009349. doi: 10.1371/journal.ppat.1009349 (PMC7963093; doi:10.1371/journal.ppat.1009349)
Supplement: Attachment — Submitted filename: Reviewer 1 Response Jan 2021 .pdf [file ppat.1009349.s010.pdf]

Professor Paul Moss,
University of Birmingham
Edgbaston
Birmingham
B15 2TA
18th January 2021

PPATHOGENS-D-20-00867 - [EMID:97b703755fb3310f]

Dear Cicin-Sain,

We apologise for the delay in addressing the point raised by reviewer 1 in response to our previous revision of the manuscript. We were awaiting delivery of reagents and ongoing CoVID pressures delayed our ability to address this point.

Reviewer #1: *The authors have done a largely good job addressing my initial concerns. I only ask that they provide evidence that their experiments using the anti-PD1 blocking antibody actually neutralized signaling in their system, as the experiment I had asked for was to include an anti-PD1 agonist, not to block PD-1 as they have in their new experiments presented in Fig 3F.*

Thank you for this. We have addressed this question utilising a Pathhunter® PD-1 (SHP1) signalling bioassay kit (Eurofins) into which we have titrated PD1-blocking antibodies. This assay contains Jurkat cells transfected with a PD-1 SHP-1 luciferase reporter construct.

Incubation of PD-L1+ target cells with Jurkat PD-1 SHP-1 cells leads to luciferase production measured by luminescence and reported as relative light units (RLU). The ability of anti-PD1 antibodies to block this signalling can then be assessed. We utilised two different anti-PD-1 antibodies, clone EH12.2H7 which was previously used in our blocking experiments, and also the Nat-105 clone. As shown below, PD-1 signalling is effectively inhibited in the presence of PD-1 blocking antibodies. Importantly, this occurs at **concentrations of antibody markedly below those used in our experiments [20µg/ml].** These data are also consistent with available data from the supplier (Biolegend). We are therefore confident that PD-1 signalling is indeed negated in our assays.

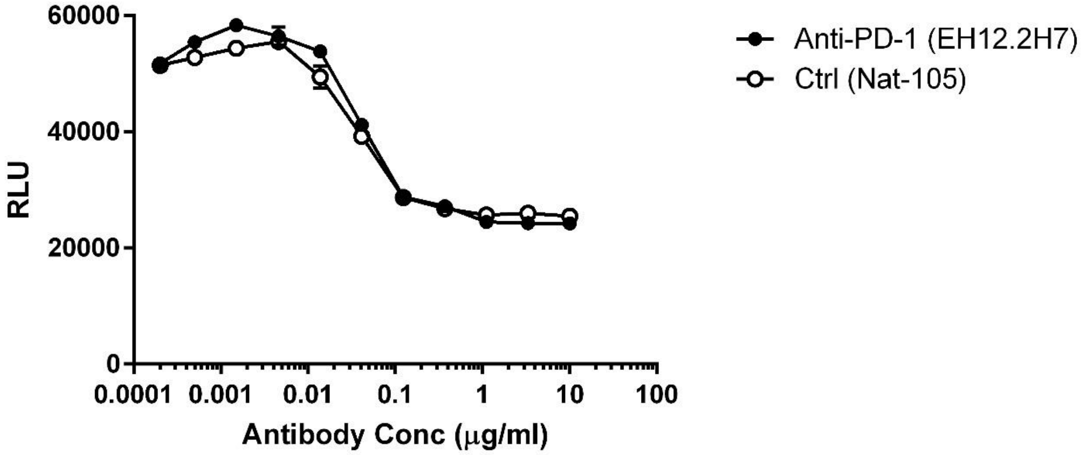

Figure. Inhibition of PD-1 signalling in Jurkat-SHP1 reporter T cells using anti-PD-1 blocking antibodies.
