# Supplementary material for: PD-1 is imprinted on cytomegalovirus-specific CD4+ T cells and attenuates Th1 cytokine production whilst maintaining cytotoxicity
Source: PLoS Pathog. 2021 Mar 4;17(3):e1009349. doi: 10.1371/journal.ppat.1009349 (PMC7963093; doi:10.1371/journal.ppat.1009349)
Supplement: Attachment — Submitted filename: Parry et al. Response to Reviewers.docx [file ppat.1009349.s009.docx]

Professor Paul Moss,

University of Birmingham

Edgbaston

Birmingham

B15 2TA

30^th^ September 2020

PPATHOGENS-D-20-00867 - [EMID:97b703755fb3310f]

Dear Cicin-Sain,

Thank you for the comprehensive and inciteful reviews of our Manuscript. The Reviewers have raised a number of important points and we are delighted to have been offered opportunity to address the comments. We have performed a wide range of additional studies and have detailed our responses below. We also attach our manuscript with amendments marked.

**Part I - Summary**

Reviewer #1: This paper from Moss and colleagues uses HLA-II tetramers recently generated by their group to characterize the PD-1 expression by HCMV epitope-specific CD4 T cells isolated from peripheral blood of normal healthy donors, or persons undergoing a primary CMV infection upon transplant. They report that PD-1 is expressed on a subset of these cells, that the initial viral load in viremic transplant patients likely regulates the setpoint of PD-1 expression which then remains stable over time, that PD-1+ cells produce less effector cytokines than their negative counterparts when restimulated ex vivo, that this diverse PD-1 expression may or may not depend upon initial CD4 T cell priming based on TCR sequencing/clonotyping, and that these PD-1 subsets show distinct RNA transcriptomes. This work advances our understanding of how PD-1 expression by HCMV CD4 T cells correlates with CD4 T cell phenotype, and with some additional experimentation showing how these phenotypes may be tied directly (or not) to PD-1 mediated signaling could be a significant advance for the field.

Reviewer #2: Parry and colleagues studied the role of PD-1 on cytomegalovirus (CMV)-specific T cells using class II tetramers to identify the cells and combine this with cell surface marker expression. Moreover, phenotypical and functional assays were performed and showed that the PD-1+ CD4 T cells are equally cytotoxic but produce lower levels of IFN-g/TNF as compared to PD-1- CD4 T cells. Thus far, little is known about PD-1+ CD4 T cells during CMV infection.

Reviewer #3: In the manuscript by Parry et al the expression of the inhibitory receptor PD-1 on Human Cytomegalovirus specific CD4+ T cells (identified by MHC Class II peptide loaded tetramers) is investigated. The manuscript considers expression following primary infection, the effect on virus load, the stability of expression over time, TcR usage in PD-1 +/- antigen specific CD4 T cells as well as effector functions and gene expression profiles by RNA-seq.

The work presented is an observational study – key findings are that there is a stable set point for proportions and antigen specific cells that express PD-1 that depending on antigen specificity the same TcR clonotypes clonotype can be represented in PD-1 +/- subsets and that these cells do not express many hallmarks associated with “exhaustion”.

A general point which should be made clear in the initial results is that the tetramers available for this work are very limited in terms of the HCMV proteins that are recognized by CD4+ T cells, 1 from gB and 2 from pp65. As such some caution should be exercised in the discussions and conclusions drawn more generally.

***Thank you for this point. We have now made amendments to the manuscript to make this clear: “Tetramers were available against three immunodominant epitopes, two from pp65 and one from gB, although it must be noted that these only identify a proportion of the global CMV-specific CD4+ T cell response”***

However, in general the manuscript is well written, the data is clearly presented, and the analysis and methods used appropriate. The overall conclusions are supported with some caveats required.

**Part II – Major Issues: Key Experiments Required for Acceptance**

Please use this section to detail the key new experiments or modifications of existing experiments that should be absolutely required to validate study conclusions.

Generally, there should be no more than 3 such required experiments or major modifications for a "Major Revision" recommendation. If more than 3 experiments are necessary to validate the study conclusions, then you are encouraged to recommend "Reject".

Reviewer #1: -The authors raise the hypothesis in the discussion and in Fig7 that perhaps PD-1+ CD4 T cells evolve to kill/clear CMV infected cells in the absence of robust inflammatory cytokine expression (e.g. IFNg), in particular they point to PD-L1+ endothelium and maintenance of vascular integrity. This is an intriguing idea, but if correct may then require a role for PD-L1/PD-1 signaling in the modulation of CTL activity or suppression of cellular signaling molecules in these CD4T cells. The authors saw no difference in cytotoxic molecules or direct killing of peptide-pulsed targets for PD-1+ or (-) cells in Fig 3DE. This peptide killing assay needs to be performed including a PD-1 agonist antibody to see if this enhances the CTL activity of the PD-1+ subset. Additionally, the activation/phosphorylation of key STAT or other TCR proximal adaptors needs to be examined in the PD-1+ and (-) cells where IFNg and TNF were only assessed in the presence of a PD-1 agonist mAb. Additionally, the authors need to test whether autologous APC infected with HCMV are more or less sensitive to killing by PD-1+ or (-) subsets, or produce different effector cytokines, as this is the real test, not the ability to kill uninfected peptide pulsed cells. There are many intriguing observations in this paper, but more direct evidence that these PD-1+ and (-) expressing CD4 T subsets mediate distinct effector functions is needed to supports the physiological importance.

***Thank you for highlighting this. We have now examined the killing of target cells by directly ex vivo isolated tetramer+ CD4 T cells in the presence or absence of PD-1 blockade. Importantly, PD-1 blockade did not increase cytotoxic activity, consistent with there being no difference in cytotoxic function between PD-1+ and PD-1- subsets. This is now included in Figure 3.***

***The lysis of virally-infected cells is an important aim for future studies but the optimisation of these assays was beyond the timeframe available for this revision in the current Covid-restricted laboratory environment. As such we have inserted the following text to make clear that further in vitro, and potentially in vivo, assays will be of interest: “Investigation using appropriate in vitro and in vivo models will be required to explore the functional importance of PD-1 expression further.”***

*The investigation of how PD-1 engagement modulates proximal TCR signalling pathways is also an excellent idea for future investigation and could represent an important report. As such we have added a comment in the discussion to highlight planned future work and to acknowledge the reviewer’s comment. “Of note, these cells provide an opportunity to study proximal aspects of TCR signaling of peptide-specific cells in relation to PD- engagement”.*

-Although there is significant evidence from many groups that CMV CD4 T cells can be cytolytic, many in the field still debate the importance/relevance of this as compared to classic CD8 CTL. Therefore, it would be extremely valuable if the authors could show how the absolute expression levels of perforin and gzm reported for CD4 tet+ cells in Fig 3D compare to a canonical HCMV-specific CD8 T cell from the same donor (e.g. pp65 or IE1 specific). Going a step further and comparing their relative per-cell killing potential compared to a HCMV CD8 T would be great in this regard as well. Even if the authors find these HCMV CD4T are not as ‘good’ as CD8 in their killing assay, or don’t express as high levels of classical cytotoxic effector molecules, this would not diminish the importance of their results in this reviewers mind. It would simply give the field a relative feeling for how to think/compare these two importance T cell effector subsets in the context of CMV infection.

***It is indeed noteworthy that most CMV-specific CD4+ T cells express strong cytotoxic activity and this is arguably the clearest example of this CD4+ phenotype in any virus-specific population. We would refer the reviewer to our previous work which characterised this phenotype in more detail (Pachnio et al, 2016) and a range of additional references in that manuscript. The comparative cytotoxicity of CD4+ and CD8+ populations is an interesting general question, somewhat outside the focus of this manuscript, but our impression is that CD4+ cytotoxic cells are at least effective in lytic capacity as CD8+ cells on a cell-to-cell basis. This will be interesting to assess in future work.***

-When analyzing HCMV-specific CD4 T cell using HLA-II tetramers, the authors only do a ‘single-staining’ analysis, using the tetramer labeled with only a single fluorophore (as shown in Fig 1A). As has been shown by the Jenkins group and others, this results in the detection of a significant number of false-positive cells which bind the tetramer non-specifically. Therefore, it is becoming the near-standard now to label the same tetramer with two separate fluorphores, and also include a 3rd non-specific tetramer (e.g. CLIP) labeled with a 3rd fluorphore, and then only identify cells that are double+ for the HCMV tetramer and negative for the CLIP as HCMV epitope-specific CD4 T cells. As most all of the authors conclusions are based on this tetramer staining analyses, it is critical that they repeat at least a few of their experiments using this 3-tetramer staining method to verify that the proportions and phenotypes of PD-1+ and PD-1(-) cells don’t change significantly. In addition, again in Fig 1A, there is no clear distinction between PD-1+ and (-) cells in the total CD4 T population. Certainly there is no clear PD-1 hi vs lo populations as you see in exhausted CD4 or CD8 T cells in tumor studies (as noted in their discussion). A bit more clarity is needed on how the authors ascribe a cell being PD-1+ or (-) to define their percentages, as no gate is shown in 1A? For instance, showing how they gated on + vs (-) cells for their RNAseq experiments.

***Thank you for this. We agree that we need to improve the description of background staining and have included an additional plot in Fig1a which shows the FMO control and gating of PD-1 in tetramer-stained samples. We have also introduced supplementary figure 2 which shows the full gating strategy for sorting of PD-1 subsets used for sequencing experiments.***

***We have also incorporated the use of a negative-control tetramer. As shown in supplementary figure 1 the level of non-specific binding is generally low and, importantly, this does not significantly alter the percentage expression of PD-1 expression in each assay. We have inserted the following text into the manuscript. “A low level of non-specific tetramer binding was revealed through the use of control tetramers but had no influence on the percentage expression of PD-1 (Sup Fig 1).”***

Reviewer #2: - In 1C the proportion of Tet+ CD4 T cells expressing one or more inhibitory receptors are plotted in bar graphs but it is complete unclear what the percentages of the individual inhibitory receptors are.

***Thank you for highlighting this and we agree that this could be considered ambiguous for the reader. We have now amended the figure to show the expression of PD-1 or other inhibitory receptors (Figure 1C) as well as the expression of these receptors on PD-1+ cells (Figure 1D).***

-In 2A a correlation of PD-1 on the CMV-specific CD4 T cells with the peak viral load is suggested based on 5 samples. The data is interesting but more data should be added.

***We agree that 5 samples are a limited data set and would like to have included further patient samples. We do have access to a large bank of transplant samples but clinical practice is to avoid transplantation of CMV- donor into CMV+ recipient due to the lack of transferred protective immunity. This, together with need for consideration of HLA genotype and availability of samples at appropriate time point, was such that appropriate samples were uncommon and we have analysed all samples for which we have access. We appreciate that this is a limited number of patients and have added additional text to the manuscript. “These data are from a limited number of patients, who are also immunosuppressed at time of infection, but may suggest that the size of the PD-1+ T cell pools expands in proportion to initial viremia whilst the PD-1- effector pool reflect a more stable baseline response following infection. Future studies will be required to further assess these findings following natural infection of healthy individuals”.***

-What is the correlation between PD-1 on the CMV-specific CD4 T cells and the percentage of the CMV-specific CD4 T cells. Likewise what is the correlation between the peak viral load and the percentage of the CMV-specific CD4 T cells.

***Thank you. This information is shown below.***

-In the same line and based on the possible like between PD-1 and CD28 expression; is there a correlation between CD28 expression on the CMV-specific CD4 T cells with peak viral load and/or percentage of CMV-specific CD4 T cells.

***These are important questions. We have now provided assessment of these relationships in supplementary figure 3.***

-2E/Fig3. CD28- are considered as cytotoxic CD4+ T cells and the PD-1+ T cells have lower expression of CD28. Still, a comparable cytotoxicity is shown in Fig 3. Do the authors have an explanation for this?

***CD28 loss is normally associated with increased differentiation towards effector memory T cells, and typically correlated with acquisition of 4-1BB expression. These data are indeed of interest and indicate that cytotoxic function is not dependent on CD28 expression in this assay. These data relate to comments in our text such that “Relative retention of CD28 suggests that a further important role for PD-1 expression may relate to relative limitation in the degree of differentiation of CMV-specific T cells and prevention of terminal exhaustion”***

-In Figure 4 it is shown that the PD-1+ CD4 T cells produce lower amounts of IFN-g and TNF. Is this also found for IL-2? The latter is of interest since IL-2 is known as the most prominent cytokine to be negatively influenced by PD-1.

***This is an interesting point, to address this we have repeated these experiments with the inclusion of staining for IL-2. However, we did not detect IL-2 production in these experiments and these data are consistent with results from other experimental methods which we have previously performed using additional epitopes. Therefore, we have added an addition note to the manuscript to indicate IL-2 is not produced by the specific cells of interest.***

***“In particular, IFN-γ and TNF-α production was observed in 42% and 43% of PD-1 cells respectively, compared to only 11% and 7.4% of the PD-1+ pool (Fig. 4b). IL-2 was not produced significantly by either PD- 1+ or PD-1- CMV-specific cells (Fig. 4c, d).”***

-The RNA sequencing data in fig 6 indicates that certain transcripts are lower in PD-1+ CD4 T cells while other are higher. The authors wrote that 10 genes were upregulated in PD-1+ cells and 5 genes were downregulated in PD-1+ cells. However, the volcano plot show that 4 genes were downregulated and 11 genes upregulated. Importantly, there is no validation of these results (except for CD28 shown in Fig 2). RT-PCR and/or protein expression studies could be done to validate at least more than 1 hit.

***Thank you. We have now amended the text to indicate that 4 and 11 genes are altered. We have also validated genes which are shown to be altered in the volcano plot by flow cytometry and these data are now shown in supplementary figure 7A.***

Reviewer #3: Figure 2

(A) I think that the authors need to interpret this result with much more caution. 3 patients have very low peak loads 2 of which have over 50% of tetramer cells PD-1 + , while 1 patients has a peak 60.10e3 but only a slightly higher % of PD-1 + cells and the other is indeed higher again. In addition these are immunosuppressed transplant patients and indeed the authors do make the point that PD-1 expression is affected in this group later on in the results section. The authors should discuss this and that data from primary infection (I appreciate this is hard) but maybe kidney transplant primary infection which has a much lower immune suppression regime could help support this hypothesis.

***Thank you. We have modified the text as highlighted below. We agree that kidney transplant patients provide an important opportunity to study this aspect of primary infection. We are currently obtaining ethical approval to recruit future kidney transplant patients in order to investigate the primary immune response in this setting in full.***

***“…correlated with the peak level of viremia at the time of primary infection. However, these data are from a limited number of patients who were also immunosuppressed at time of infection. As such these data may suggest that the size of the PD-1+ T cell pools expands in proportion to initial viremia whilst the PD-1- effector pool reflect a more stable baseline response following infection, further studies will be required to assess these findings in a setting of reduced immunosuppression, or ideally natural infection of healthy individuals.”***

Figure 4 attenuation of cytokine production in PD-1 = T cells.

LCL cells lines which are EBV transformed have been used as antigen presenting cells (as they express PD-L1) in these experiments pulsed with CMV peptides and co culture with whole PBMC. The readout is intracellular IFN gamma or TNFa – however this would be measuring T cell responses to not only the HCMV peptides but also to EBV presented antigens – as such the results between PD-1 +/- are a mixture of T cells specific to 2 different herpesviruses. However, in the figure legend it states that these were HCMV tetramer positive cells, which would be a much more logical experiment. Could the authors please clarify the experiment and have the results and figure legend match.

***Thank you. The LCLs used in these assays were partially matched to the appropriate HLA-allele of the CMV epitope of interest. Thereby limiting recognition of EBV epitopes. Additionally, as correctly noted the PBMC were co-stained with tetramer allowing identification of the relevant population. To make this clear we have updated the text and legend;***

***Text: incubated with peptide-pulsed lymphoblastoid target cells. PBMC were then co-stained with CMV specific tetramer and the profile of cytokine production determined by intracellular flow cytometry.***

***Legend: Whole PBMCs were stimulated with HLA-matched, peptide pulsed LCLs and IFN-γ and TNF-α release was measured in*** ***CMV tetramer+ CD4+ T cells by intracellular staining. (A) An example showing CMV tetramer+ CD4+ T cell cytokine expression versus PD-1. (B) Pooled data of the frequency of cytokine expression in CMV tetramer+ CD4+ T cells in healthy donors (n=7).***

In the blocking experiments CMV lysate is used to pulse whole PBMC given the previous experiment used LCLs as they had PD-L1 expression, what is the expression of PD-L1 on subsets in PBMC. The effect of blocking antibody in this experiment shows good results in only 3 of the donors tested while 4 others have minimal increases in IFN g? The authors should address these points.

***Thank you. We have now assessed the expression of both PD-1 ligands in whole PBMC, as shown in Supplementary figure 5. Expression of PD-L1 was predominantly detected in monocyte and B cell fractions subsets. However, the level of expression remained consistent across the donors tested, indicating that this is unlikely to be the mechanism governing the level of response in previous assays.***

“The response of individual donors to PD-1 blockade was variable but did not appear related to the presence of PD-1 ligands which was consistent across individuals (Sup Fig 5.)”

Part III – Minor Issues: Editorial and Data Presentation Modifications

Please use this section for editorial suggestions as well as relatively minor modifications of existing data that would enhance clarity.

Reviewer #1: -Can the authors please describe the method for how the limit-dilution cloned PD-1+ and (-) cells that were FACS sorted were kept alive for 4 weeks to assess the maintenance of PD-1 expression. Were they restimulated weekly with autologous APC+peptide (LCL in methods?)…IL-2..something else?

***We have updated the method section to provide further details of the procedures used to generate and maintain these T cell clones.***

***“CMV-specific CD4+ T cells by limited dilution cloning as previously described (https://pubmed.ncbi.nlm.nih.gov/21622860/). These were then grown in T cell media (RPMI-1640 supplemented with 10% fetal calf serum, 1% human serum, 50U/ml IL-2, 30% MLA and 100IU/ml Penicillin and 100μg/ml Streptomycin).***

***Clones were generated and re-stimulated as required by addition of 1x105 peptide pulsed LCL and 1x106 irradiated PHA activated PBMC. Lymphoblastoid cell lines (LCL) were generated as previously described by transforming B cells derived from donors of known HLA type (13).***

***LCL were incubated for 60 minutes at 37oC with the appropriate peptide at 1μg/ml. Cells were then washed twice and resuspended in T cell media.”***

***We have updated the methods section and the text in the results section to clarify the simulation of clones over the period PD-1 levels were measured weekly.***

***Results “4-week period following stimulation (at day 0), no further stimulation was provided over the culture period (Fig. 2D)”***

***Method “For experiments studying the retention of PD-1 by T cell clones, re-stimulation (as above) was defined as day 0, no further stimulation was provided. Clones were fed twice weekly by replacement of half culture volume with T cell media.”***

-The authors show in Fig 1D that a small % of tet+ HCMV CD4T also express one or more other markers which are often used to identify ‘exhausted’ T cells. The raw FACS histograms where they gated/identified these cells as + for these other markers needs to be shown, because small increases in MFI would not be consistent with the conclusion that these cells are more exhausted, as this is a similar case to PD-1+ vs +++ for instance.

***Thank you for highlighting this. We have now included histogram plots to show these additional inhibitory markers (supplementary figure x)***

Reviewer #2: The authors should better indicate which groups are plotted in 1B (e.g. Tet+ and Tet- could be used instead of Tet and CD4).

***We have amended Fig 1B to show Tet+/-.***

Reviewer #3: End of first results section “…CD4+ T cells is not related to activation status or exhaustion.” Could you amend this to “…CD4+ T cells is not related to markers of activation status or markers of exhaustion.”

***We have amended the text to “is not related to markers of activation or exhaustion.”***
